# A mathematical modeling technique to understand the role of decoy receptors in ligand-receptor interaction

**DOI:** 10.1038/s41598-023-33596-z

**Published:** 2023-04-21

**Authors:** Subrata Dey, Aditi Ghosh, Malay Banerjee

**Affiliations:** 1 Department of Mathematics and Statistics, Indian Institute of Technology Kanpur, Kanpur, Uttar Pradesh India; 2 Texas A&M University Commerce, Commerce, 75429 TX US

**Keywords:** Applied mathematics, Cell biology

## Abstract

The ligand-receptor interaction is fundamental to many cellular processes in eukaryotic cells such as cell migration, proliferation, adhesion, signaling and so on. Cell migration is a central process in the development of organisms. Receptor induced chemo-tactic sensitivity plays an important role in cell migration. However, recently some receptors identified as decoy receptors, obstruct some mechanisms of certain regular receptors. DcR3 is one such important decoy receptor, generally found in glioma cell, RCC cell and many various malignant cells which obstruct some mechanism including apoptosis cell-signaling, cell inflammation, cell migration. The goal of our work is to mathematically formulate ligand-receptor interaction induced cell migration in the presence of decoy receptors. We develop here a novel mathematical model, consisting of four coupled partial differential equations which predict the movement of glioma cells due to the reaction-kinetic mechanism between regular receptors CD95, its ligand CD95L and decoy receptors DcR3 as obtained in experimental results. The aim is to measure the number of cells in the chamber’s filter for different concentrations of ligand in presence of decoy receptors and compute the distance travelled by the cells inside filter due to cell migration. Using experimental results, we validate our model which suggests that the concentration of ligands plays an important role in cell migration.

## Introduction

Cell migration in unicellular and multi-cellular organisms is an evolutionary conserved mechanism that contributes to their normal development and various cell functioning such as embryogenesis, wound healing, immune responses, cancer metastases, and angiogenesis^1^. It is influenced mainly by two mechanisms: (a) chemokinesis, which corresponds to the random movement of cells, (b) chemotaxis, which refers to the directed movement of cells either toward attractant or away from repellent chemicals. In cellular biology, the ligand-receptor interaction plays a significant role in chemotaxis^2^. Apart from its chemokinesis random locomotion, cells can move towards or away from extracellular complex produced through interaction of receptor and ligand/chemical^2,3^. Chemotaxis due to interaction of ligand, normal and decoy receptors have been widely studied in the field of biology but cell migration due to the inclusion of decoy receptors in presence of normal receptors and ligand has not been mathematically formulated and experimentally validated^4,5^. Cell migration due to chemotaxis which has widespread application in immunotaxis, leukocyte migration during immune response to inflammation involves complex molecular biology^6,7^. Although much study has been conducted in understanding the role of decoy receptors and the associated biochemical responses, there is a critical need to interpret the responses of these biological processes involved in chemotaxis through quantitative predictions. Thus a ready to use mathematical model plays a significant role in formulating cell migration due to ligand-receptor interaction and validating it experimentally to predict the dynamics of interaction of normal receptor CD95, decoy receptor DcR3 and ligand CD95L^8^. Thus we develop here a mathematical model the novelty of which captures the influence of decoy receptors in cell migration due to receptor-ligand interaction.

Receptor is a composition of protein that is usually distributed in the cell membrane. The largest family of receptors is G protein-coupled receptors^2^. An intracellular receptor interacts with a variety of ligands, including hormones, neurotransmitters, oxygen, and glycoproteins. As a result of the interaction, the receptor conforms to stabilize or induce the activation of a heterotrimeric G protein (consists of $\alpha$, $\beta$, and $\gamma$ subunits) on the inner membrane surface of the cell^9,10^. In the inactive heterotrimeric state, guanosine diphosphate (GDP) is bound to the G$\alpha$-subunit. Upon activation, GDP is released, guanosine triphosphate (GTP) binds to G$\alpha$, and subsequently, G$\alpha$-GTP dissociates from G$\beta \gamma$ from the receptor^10,11^. Both G$\alpha$-GTP and G$\beta \gamma$ are then free to activate downstream effectors^10,11^ which is responsible for cell signaling.

Cell signaling through ligand-receptor interaction occurs when regulatory chemical or ligand (*c*) bind with the receptor on the cell surface^12^. The bound receptors can be recycled back to the cell surface after cell signaling. The reaction mechanism between free receptors $R_f$ and *c* behind the cell signaling is as follows^2,13^

1 $$\begin{aligned} R_f+c\mathop {\rightleftharpoons }\limits _{k_{d}}^{k_{a}} R_b\; \xrightarrow {k_{i}} \text {Internalized receptor complex chemical}, \end{aligned}$$

where $R_{b}$ is the bound receptor. The bound receptor $R_b$ modulates pseudopod extension^4,5^ which is critical in sensing the direction of the movement.

Decoy receptors are some special type of receptors that acts as an inhibitor in cell signalling induced by normal receptor. IL-1R2 was identified as the first decoy receptor in the ’90s^14^. Most of the mechanisms of the decoy receptors are yet to be known in the view of cellular biology^15,16^. The subfamily of decoy receptor includes DcR3^17^, D6^18^, Duffy Antigen Receptor for Chemokines (DARC)^19^, etc. Decoy receptor 3 (DcR3)^17,20^, a well-known member of tumor necrosis factor receptor superfamily (TNFRSF), is capable of neutralizing the biological effect of other three tumor necrosis factor superfamily (TNFSF) members, namely Fas ligand (FasL/CD95L)^21^, LIGHT^22^ and TL1A^23^. Based on its neutralizing effects on CD95L, LIGHT, and TL1A, DcR3 can be defined as an immunomodulator^17,20^, since CD95L and LIGHT induce apoptosis and inflammation, and TL1A is a T cell costimulator. DcR3’s overexpression was found in various malignant cells such as RCC cell^24^, Gliomas cell^25^, Glioblasomas cell^26^. Experimental findings by Weissinger et al.^25^ and Roth et al.^8^ suggest that DcR3 binds with CD95L and neutralizes CD95L chemo-tactic activity. As a result, cell migrated area and cell motility were decreased in the presence of DcR3. The decoy receptor’s interaction with ligand was captured in a mathematical model in^27^, but the chemo-repellent activity of the bound decoy receptors does not influence the cell migration in that model nor the application of the model is explored well in^27^. These complicated facets of cell motility are exclusively investigated in this framework of partial differential equations. For instance, we measure the migrated cell density in the Boyden chamber’s filter for different concentrations of ligand and compute the distances travelled by the cells inside filter. In particular, we investigate mathematically how the cell migration is influenced by the interaction of normal receptors CD95L, decoy receptors DcR3 and ligand CD95L via a mathematical model.

Chemo-taxis and Brownian motion are used to model chemically modulated cell motility following the pioneering work of Kellar & Segal^28^. In^28^, the authors have considered cell-flux at spatial point *x* and at an instant of time *t* ($J_n(x,t)$) which is dependent on cell density *n*(*x*, *t*) and chemical concentration *c*(*x*, *t*) through the following equation

2 $$\begin{aligned} J_n(x,t)=-D_n \frac{\partial n(x,t) }{\partial x}+\chi _n(c) n(x,t)\frac{\partial c(x,t)}{\partial x}, \end{aligned}$$

where $D_n$ represents random movement of cell and $\chi _n(c)$ represents the chemo-tactic sensitivity, which is chemo-attractant if $\chi _n(c)>0$ for all *c* and chemo-repellent if $\chi _n(c)<0$ for all *c*. Since chemo-attractants or chemo-repellents activate cell’s surface receptors, it is interesting to explore how the receptor-ligands interaction affects the motility of cells. Sherratt et al.^13^ have proposed a three compartment model of cell *n*, ligand *c* and bound receptors $\rho$ with some modification of cellflux of (2) to account the receptor-ligand mechanism. Despite the importance of these research and the crucial outcome they provided, their work did not account for cell migration due to decoy receptors. In this study, we have incorporated decoy receptors as chemo-repellent and chemo-attractant to elucidate the effect of these receptors in cell migration. We assume that extracellular complex produced through interaction of receptor and ligand attracts the cells whereas the ligand-decoy complex repeals. These two reaction occurs simultaneously through multiple receptor state in Boyden chamber.

The Boyden chamber^3,29^ has become a popular tool to estimate the coefficients of random cell walks and chemo-tactic behaviors for various cell types by fitting the experiments with theoretical computations in vitro system. It is classically constructed with two wells separated by a porous filter, which acts as a physical barrier and active migration is the only way to overcome it. A chemo-attractant solution is placed in the lower well while a cell suspension is placed in the upper well. Cells are allowed to migrate inside the filter in response to the chemical distribution for a given time interval. Cell motility is measured from the distance that cell wave fronts reach inside the filter during the incubation time. Sherratt et al.^13^ have shown the movement of cells in filter affected by the normal receptor in Boyden chamber. Chen et al.^30^ studied cell migration due to receptor-ligand interaction with cell sedimentation in Boyden chamber. Zigmond and Dunn chambers are two other chemotaxis bridge assays which studies cell motility of polymorphonuclear (PMN) leukocytes and fibroblasts respectively. The Zigmond chamber with efficient optical properties generates shallow gradients so that cells could respond to only 1% changes in concentration^31^. The Dunn chamber studies the migration of fibroblasts whose migration rate is $0.42-1.25\,\upmu \hbox {m}/\hbox {min}$ and generates a linear gradient within an hour of its initial setting with a gradient half-life of 10 to 30 hours. The Insall chamber a refinement of Dunn chamber, consists of two-well bridge design assay with defined directions of gradient and two different gradient steepness in the same assay. It measures melanoma chemotaxis^31^.

In this work, we mathematically formulate the effect of cell migration in presence of decoy receptors and normal receptors. To the best of our knowledge, no mathematical model has considered cell migration caused by decoy receptors and studied chemo-repellent activity of the bound decoy receptors. We construct a novel mathematical model to study and validate the movement of glioma cells in presence of normal receptors CD95, ligand CD95L and decoy receptors DcR3 mathematically. The novelty of our mathematical model is the inclusion of decoy receptors in cell migration. The goals of our work are to: (i) propose a new mathematical model to understand the role and dynamics of decoy receptor DcR3 in cell migration based upon the classic Keller-Segel model^28^, (ii) study the system dynamics in presence and absence of decoy receptors DcR3, (iii) measure the migrated cell density in the Boyden chamber’s filter for different concentrations of ligand in response to chemo-tactic sensitivity of normal and decoy receptors, (iv) compute the distances travelled by the cells inside filter due to cell migration. Here, we consider both decoy receptors as well as normal receptors present for cell migration. We fill the upper well and lower well with cells and ligands respectively and study the dynamics of the system in filter compartment.

## Theoretical study of receptor-ligand modeling

### Governing equations

**Figure 1:**
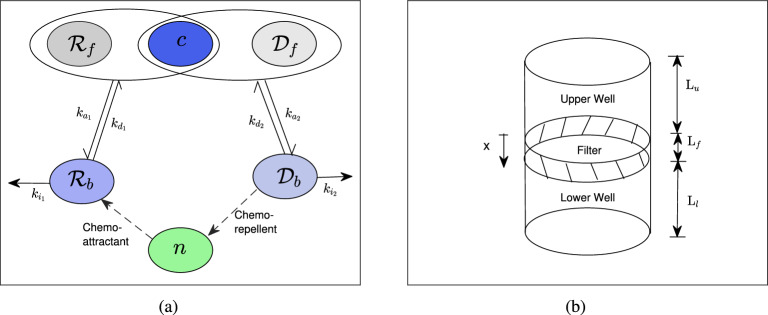
(**a**) Schematic presentation of cell migration in response to receptor-ligand interaction. Solid arrow indicates the conversion of the same variable according to the system (3) and dashed arrow represents the chemo-tactic sensitivity of the bound receptors. (**b**) Schematic representation of the Boyden chamber. A filter separates the chamber into two wells. The upper well and lower well are filled with cells and chemicals or ligands respectively. In response to chemo-tactic sensitivity, cells settle onto the filter and migrate through it.

We consider the normal receptor-ligand and decoy receptor-ligand interaction to occur simultaneously as per multiple receptor states^2^. Assume that the normal receptors $\mathscr {R}_f$ and decoy receptors $\mathscr {D}_f$ bind with the ligands at a rate $k_{a_1}$ and $k_{a_2}$ and form the bound normal receptors $\mathscr {R}_b$ and bound decoy receptors $\mathscr {D}_b$ respectively. Let the degradation rate of $\mathscr {R}_b$ and $\mathscr {D}_b$ are $k_{d_1}$ and $k_{d_2}$ respectively. The bound normal receptor $\mathscr {R}_b$ ($\mathscr {D}_b$) turn into internalization components at a rate $k_{i_1}$ ($k_{i_2}$). The flowchart of the normal and decoy receptor-ligand interaction is shown in Fig. 1a. Using the law of mass action, the pathophysiology is modeled by the following system of Eq. (3)

3a $$\begin{aligned} \frac{d c}{d t}&=k_{d_1} \mathscr {R}_b-k_{a_1} c \mathscr {R}_f+k_{d_2} \mathscr {D}_b-k_{a_2} c \mathscr {D}_f,{} & {} \end{aligned}$$

3b $$\begin{aligned} \frac{d \mathscr {R}_b}{d t}&=k_{a_1} c \mathscr {R}_f-\left( k_{d_1} +k_{i_1}\right) \mathscr {R}_b,{} & {} \quad \quad \frac{d \mathscr {R}_f}{d t}=k_{d_1} \mathscr {R}_b-k_{a_1} c \mathscr {R}_f, \end{aligned}$$

3c $$\begin{aligned} \frac{d \mathscr {D}_b}{d t}&=k_{a_2} c \mathscr {D}_f-(k_{d_2} +k_{i_2})\mathscr {D}_b,{} & {} \quad \quad \frac{d \mathscr {D}_f}{d t}=k_{d_2} \mathscr {D}_b-k_{a_2} c \mathscr {D}_f. \end{aligned}$$

subject to non-negative initial conditions. Note that the net mass-balance of system (3) is negative due to the internalization of bound receptors and decoy receptors^2,13,32^. The fate of bound receptors can vary depending on several factors, including the receptor type, the nature of the ligand, and the number of times they have interacted. In some cases, the internalized receptors can be recycled back to the cell surface as free receptors with the ligand, allowing for continued signaling. However, in other cases, the bound receptors may be targeted for degradation in lysosomes through internalized endocytosis^2,30^, which can lead to decreased signaling and modulation of cellular responses.

To study chemo-tactic sensitivity of the normal receptors and decoy receptors, we spatially extend system (3) by considering the Boyden chamber as spatial domain. The Boyden chamber (see Fig. 1b) consists of three compartments namely, upper well, lower well and filter. We fill the upper well and lower well with cells and ligands respectively. The movement of the cell depends on the interaction of ligands and normal receptors/ decoy receptors which are situated at the surface of the cell. Neglecting the radial variation, we consider the one-dimensional geometry of Boyden chamber as the domain for our spatio-temporal model. Let the thickness of lower well, filter and upper well be $L_l$, $L_f$ and $L_u$ respectively. We consider the upper surface of the filter as $x=0$. Chemical *c* is defined on $-L_u<x<L_l+L_f$ as it can diffuse throughout the chamber. We are interested to understand the movement of cells in the filter only in presence of decoy and cell receptors. There is an inflow and outflow of cells in the upper and lower surface of filter respectively. Chemicals can diffuse throughout the chamber with no-flux boundary conditions at both end of the chamber. The bound receptors and bound decoy receptors on the cell surface act as chemo-attractant and chemo-repellent. Therefore the cell-flux is modelled by $\displaystyle K_n=-D_n \frac{\partial n }{\partial x}+\chi n \frac{\partial \rho _r}{\partial x}-\mu n \frac{\partial \rho _d}{\partial x},\;$ where $\rho _r=\left( \frac{\mathscr {R}_b}{n}\right)$ and $\rho _d=\left( \frac{\mathscr {D}_b}{n}\right)$ are the number of normal receptors and bound receptors per cell respectively. Parameters $\chi$ and $\mu$ are the chemo-tactic attractant and repellent coefficient respectively. The spatio-temporal behavior of the system in $0<x<L_f$ is given by

4a $$\begin{aligned} \frac{\partial n}{\partial t}&=-\frac{\partial K_n}{\partial x},\;{} & {} \quad \frac{\partial c}{\partial t}=D_c \frac{\partial ^2 c}{\partial x^2}+k_{d_1} \mathscr {R}_b-k_{a_1} c \mathscr {R}_f+k_{d_2} \mathscr {D}_b-k_{a_2} c \mathscr {D}_f, \end{aligned}$$

4b $$\begin{aligned} \frac{\partial \mathscr {R}_b}{\partial t}&=-\frac{\partial }{\partial x} \left( \rho _rK_n\right) +k_{a_1} c \mathscr {R}_f-\left( k_{d_1} +k_{i_1}\right) \mathscr {R}_b,\;{} & {} \quad \frac{\partial \mathscr {D}_b}{\partial t}=-\frac{\partial }{\partial x} \left( \rho _nK_n\right) +k_{a_2} c \mathscr {D}_f-(k_{d_2} +k_{i_2})\mathscr {D}_b. \end{aligned}$$

All the variable used in the system (4) is mentioned Table 1. In other compartments, we assume that chemical *c* flows through diffusion only. *c*(*x*, *t*) is following the reaction-diffusion equation

**Table 1:** Description of the variables used in the model.

Variable	Description	Unit
*n*	Cell density	Counts/cm$^3$
*c*	Chemical concentration	$\mu$g/cm$^3$
$\mathscr {R}_b$	Concentration of free normal receptors	mol/cm$^3$
$\mathscr {R}_f$	Concentration of bound normal receptors	mol/cm$^3$
$\mathscr {D}_f$	Concentration of free decoy receptors	mol/cm$^3$
$\mathscr {D}_f$	Concentration of bound decoy receptors	mol/cm$^3$
$\rho _r$	Number of moles of bound normal receptors per cell	mol
$\rho _d$	Number of moles of bound decoy receptors per cell	mol

5 $$\begin{aligned} \frac{\partial c}{\partial t}=D_c \frac{\partial ^2 c}{\partial x^2}. \end{aligned}$$

We also need the continuity of chemical flux $(\frac{\partial c}{\partial x}),$ at the both end of filter $x=0$ and $x=L_f.$

Let the diameter of the cell be *L*, then inflow and outflow cell-flux can be modelled in the boundary condition as

6 $$\begin{aligned} \frac{\partial n}{\partial t}=-\frac{K_n}{L}\;\text {for }x&=0 \text { and } \frac{\partial n}{\partial t}=\frac{K_n}{L}\;\text {for }x&=L_f. \end{aligned}$$

In the absence of ligand, a certain number of free receptors are typically present on the cell surface. However, when the ligand is present and binds to its receptors on the cell surface, it can trigger a signaling cascade that ultimately results in the upregulation of additional receptors^30,33^. This upregulation can occur through several mechanisms, including increased transcription and translation of receptor genes, decreased degradation of existing receptors, and increased recycling of internalized receptors back to the cell surface. To incorporate such upregulation mechanism, we assume that total number of free receptors and bound receptors per cell is increasing with rate $f(\rho _r)=\Gamma _r(1+\beta \rho _r),$ where $\Gamma _r$ is the initial number of moles of free receptors on a cell and $\beta$ is the increasing rate of the total receptor number on a cell surface per binding to the chemo-attractant receptor. So, the relation between receptors and bound receptors^13,30^ is given by $\displaystyle \mathscr {R}_b+\mathscr {R}_f=\Gamma _r(n+\beta \mathscr {R}_b).$ Similarly, for the decoy receptors we consider $\displaystyle \mathscr {D}_b+\mathscr {D}_f=\Gamma _d(n+\delta \mathscr {D}_b).$ These two relations reduce the number of dependent variables. Finally, system (4) can be written as

7a $$\begin{aligned} \frac{\partial n}{\partial t}&=-\frac{\partial K_n}{\partial x}, \end{aligned}$$

7b $$\begin{aligned} \frac{\partial c}{\partial t}&=D_c \frac{\partial ^2 c}{\partial x^2}+k_{d_1} n \rho _r-k_{a_1} c n \left( \Gamma _r(1+\beta \rho _r) - \rho _r\right) +k_{d_2} n \rho _d-k_{a_2} c n (\Gamma _d(1+\delta \rho _d)- \rho _d), \end{aligned}$$

7c $$\begin{aligned} \frac{\partial \rho _r}{\partial t}&=- \frac{K_n}{n}\frac{\partial \rho _r}{\partial x}+k_{a_1} c \left( \Gamma _r(1+\beta \rho _r) - \rho _r\right) -\left( k_{d_1} +k_{i_1}\right) \rho _r, \end{aligned}$$

7d $$\begin{aligned} \frac{\partial \rho _d}{\partial t}&=-\frac{K_n}{n}\frac{\partial \rho _d}{\partial x}+k_{a_2} c (\Gamma _d(1+\delta \rho _d)- \rho _d)-(k_{d_2} +k_{i_2}) \rho _d, \end{aligned}$$

All the parameters involved in the system (7) are positive and summarized in the Table  2.

**Table 2:** Description of the parameters used in the model.

Parameters	Description	Values	Sources
$D_n$	Random motility of cell	$1.3 \times 10^{-10}\text {cm}^2/\text {s}$	^13^
$D_c$	Random motility of chemical	$7.3 \times 10^{-6} \text { cm}^2/\text {s}$	^13^
$\chi$	Chemo-tactic attractant coefficient	$2 \times 10^{12} \text {cm}^2/(\text {s mol})$	^13^
$\mu$	Chemo-repellent coefficient	$10^{10}$ $\text {cm}^2/(\text {s mol})$	Estimated
$k_{d_1}$	Dissociating rate of normal receptors	0.0058 /s	^34^
$k_{d_2}$	Dissociating rate of decoy receptors	0.0029 /s	Estimated
$k_{a_1}$	Associating rate of normal receptors	$1.67 \times 10^{7}$ cm$^3$/(s $\mu$g)	^34^
$k_{a_2}$	Associating rate of decoy receptors	$8.33 \times 10^{7}$ cm$^3$/(s $\mu$g)	Estimated
$k_{i_1}$	Internalizing rate of normal receptors	0.004 /s	^34^
$k_{i_2}$	Internalizing rate of decoy receptors	$8\times 10^{-4}$ /s	Estimated
$\beta$	Increasing rate of normal receptors	(0.95-1)/$\Gamma$	Estimated
$\delta$	Increasing rate of decoy receptors	(0.95-1)/$\Gamma$	Estimated
$\Gamma _r$	Number of moles of free receptors	$4.98 \times 10^{-21}$ mol	^13^
	on cell surface in absence of chemical		
$\Gamma _d$	Number of moles of decoy receptors	$3.984 \times 10^{-21}$ mol	Estimated
	On cell surface in absence of chemical		
*L*	Diameter of cell	9.2 $\times 10^{-4}$ cm	^13^
$L_u$	Height of the upper well	0.0375 cm	^13^
$L_f$	Height of the filter	0.015 cm	^13^
$L_l$	Height of the lower well	0.03125 cm	^13^

### Non-dimensionalization

To reduce the number of parameters, we need to do non-dimensionalization. We choose the filter thickness $L_f$ as a length scale and one hour time *T* as a time scale i.e. $\tilde{x}=\frac{x}{L_f} \text{ and } \tilde{t}=\frac{t }{T}$. The cell density, chemical concentration are scaled by the initial cell density in the upper well $n_0$, chemical concentration in the lower well $c_0$ respectively. The bound normal receptor number and bound decoy receptor are scaled by the original free normal receptor $\Gamma _r$ and decoy receptor number $\Gamma _d$ respectively. So, the dimensionless variables are

$$\begin{aligned} \tilde{n}=n/n_0,\;\tilde{c}=c/c_0,\; \tilde{\rho _r}=\rho _r/\Gamma _r,\; \tilde{\rho _d}=\rho _d/\Gamma _d.\; \end{aligned}$$

The other dimensionless parameters are as follows

$$\begin{aligned} \tilde{D_n}= & {} \frac{D_nT}{L_f^2},\;\tilde{\chi }=\frac{\chi T \Gamma _r}{L_f^2}, \tilde{\mu }=\frac{\mu T \Gamma _d}{L_f^2},\;\tilde{D_c}=\frac{D_cT}{L_f^2},\;\tilde{k_{d_1}}=k_{d_1}T,\;\tilde{k_{d_2}}=k_{d_2}T,\;\tilde{k_{a_1}}=k_{a_1}Tc_0,\;\tilde{k_{a_2}}=k_{a_2}Tc_0,\\ \tilde{k_{i_1}}= & {} k_{i_1}T,\;\tilde{k_{i_2}}=k_{i_2}T,\;\tilde{\Gamma _r}=\frac{\Gamma _r n_0}{c_0},\;\tilde{\beta }=\beta \Gamma _r,\;\tilde{\delta }=\delta \Gamma _d,\;\tilde{\Gamma _d}=\frac{\Gamma _d n_0}{c_0},\;\tilde{L_l}=\frac{L_l}{L_f},\; \tilde{L_u}=\frac{L_u}{L_f},\;\tilde{L}=\frac{L}{L_f}. \end{aligned}$$

For simplicity, we drop the tilde notation. Then, the dimensionless system can be written on the filter section i.e. on $0<x<1$ as

8a $$\begin{aligned} \frac{\partial n}{\partial t}=&-\frac{\partial K_n}{\partial x}, \end{aligned}$$

8b $$\begin{aligned} \frac{\partial c}{\partial t}=&D_c \frac{\partial ^2 c}{\partial x^2}+k_{d_1}\Gamma _r n \rho _r-k_{a_1} c n \Gamma _r \left( 1+(\beta -1) \rho _r \right) +k_{d_2} \Gamma _b n \rho _d-k_{a_2} cn \Gamma _d(1+(\delta -1) \rho _d), \end{aligned}$$

8c $$\begin{aligned} \frac{\partial \rho _r}{\partial t}=&- \frac{K_n}{n}\frac{\partial \rho _r}{\partial x}+k_{a_1} c \left( 1+(\beta -1) \rho _r \right) -\left( k_{d_1} +k_{i_1}\right) \rho _r, \end{aligned}$$

8d $$\begin{aligned} \frac{\partial \rho _d}{\partial t}=&-\frac{K_n}{n}\frac{\partial \rho _d}{\partial x}+k_{a_2} c (1+ (\delta -1) \rho _d)-(k_{d_2} +k_{i_2}) \rho _d, \end{aligned}$$

where $\displaystyle K_n=-D_n \frac{\partial n }{\partial x}+\chi n \frac{\partial \rho _r}{\partial x}-\mu n \frac{\partial \rho _d}{\partial x}$, and on the other compartment of the Boyden’s chamber i.e. $\text { on } -L_l<x\le 0 \text{ and } 1\le x<L_u,$

9 $$\begin{aligned} \frac{\partial c}{\partial t}=D_c \frac{\partial ^2 c}{\partial x^2}. \end{aligned}$$

### Initial and boundary conditions

Cell migration in response to the ligand-receptor interaction induced chemotactic sensitivity is modelled here through system (8). Hence to study cell migration in various chemotactic assays such as Boyden chamber^29^, Dunn chamber^35^, Zigmond chamber^36^ etc., we need to incorporate well-defined initial and boundary conditions according to the system. Our model here is not assay specific, the boundary conditions change with different assays. In our study here, we consider the Boyden chamber’s one-dimensional geometry as a spatial domain of our system (8–9) which is solved using appropriate initial and boundary conditions. The initial cell number density is considered to be uniform in the upper well and zero in other compartment respectively and boundary condition follows Eq. (6). We also assume that there is no chemical in the upper well initially and the flux in both surfaces of the filters is continuous with no flux at both ends of the chamber. The initial and boundary conditions, in terms of dimensionless variables, can be summarized as followsInitial condition:

$$\begin{aligned} n(x,0)= & {} 0, \,x\in \,[0,L_f),\,\,n(L_f)=1;\,\, \rho _r(x,0)=\rho _d(x,0)=0,\, x\,\in \,[0, L_f];\\ c(x,0)= & {} 1, x\,\in \,[-L_l, 0],\,\, c(x,0)=0,\,x\,\in \,[0, L_f]. \end{aligned}$$

Boundary condition:

$$\begin{aligned} \begin{array}{lll} &{}\text { at } x=-L_l \text { and }L_u: \; \frac{\partial c}{\partial x}=0, &{} \quad \quad \text { at } x=0:\; \frac{\partial n}{\partial t}=-K_n/L,\; \nabla . \rho _r=0 \text { and }\frac{\partial \rho _d}{\partial x}=0,\\ &{}\text { at } x=L_f:\; \frac{\partial n}{\partial t}=+K_n/L,\; \nabla . \rho _r=0 \text { and }\frac{\partial \rho _d}{\partial x}=0, &{} \quad \quad \text {and } \frac{\partial c}{\partial x} \text { is continuous at } x=0,L_f. \end{array} \end{aligned}$$

## Results

### Sensitivity analysis

System (8) contains fourteen parameters among few parameters that were not obtained experimentally and hence we identify here significant parameters which affect the system’s dynamics the most. To determine the sensitivity of these parameters, we perform a forward sensitivity analysis of a PDE model using the approach described in^37–39^. Here we are interested to obtain the sensitivity index of the number of migrant cells in the filter $N_f$ at $\tau =5,$ where $\displaystyle {N_f=n_0\int _0^1n(x,\tau )dx.}$ Let $y=(y_1,y_2,y_3,y_4)^T=(n,c,\rho _r,\rho _d)^T,$ and $p=(p_1,p_2,p_3,p_4,p_5,p_6,p_7,p_8)^T=(\chi ,\mu ,\beta ,\delta ,k_{a_2},k_{d_2},k_{i_2},\Gamma _d)^T$. Then we can write our model (8) in a matrix form as

10 $$\begin{aligned} \frac{\partial y}{\partial t}=\mathscr {F}(y,p,y_x,y_{xx}) \end{aligned}$$

where $\mathscr {F}(y,p,y_x,y_{xx})\equiv (\mathscr {F}_1,\mathscr {F}_2,\mathscr {F}_3,\mathscr {F}_4)^T$ are the reaction terms in the right-hand side of (8). The sensitivity index $S_j$ of the parameter $p_j$ is defined as

$$\begin{aligned} S_j=\frac{p_j\int _0^1\frac{\partial y_1(x,\tau )}{\partial p_j}dx}{\int _0^1 y_1(x,\tau )dx} \;\quad \text {for }j=1,\cdots ,8. \end{aligned}$$

To find the value of $\displaystyle {\frac{\partial y_1}{\partial p_j}}$, we need to solve

11 $$\begin{aligned} \frac{\partial }{\partial t}\left( \frac{\partial y}{\partial p_j}\right) =\frac{ \partial \mathscr {F}(y,p,y_x,y_{xx})}{\partial p_j}, \end{aligned}$$

simultaneously with the system (10). More details of the method are given in the supplementary information S1.

**Figure 2:**
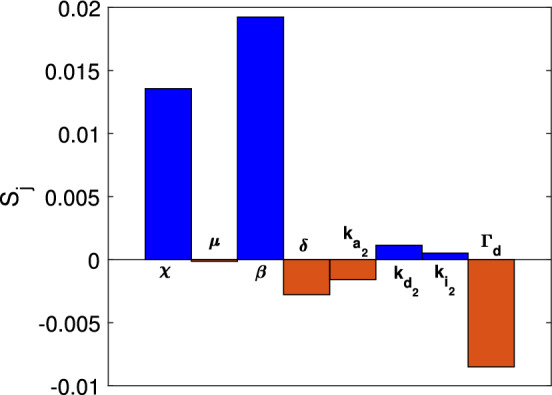
Bar diagram representing the sensitive index of eight parameters used in the model (8).

The sensitive index $S_j$ corresponding to eight important parameters namely $\chi ,\mu ,\beta ,\delta ,k_{a_2},k_{d_2},k_{i_2},$ and $\Gamma _d$ are summarized in Table 3. The positive sign of $S_j$ implies that if the corresponding parameter $p_j$ increases then $N_f$ increases and the negative sign of $S_j$ implies that if $p_j$ increases then $N_f$ decreases for $j=1,\cdots ,8.$ We also plot the bar diagram of $S_j$ in Fig. 2 which shows that $\chi ,\beta ,k_{d_2}$ and $k_{i_2}$ are positively correlated to $N_f$ whereas $\mu ,\delta ,k_{a_2}$ and $\Gamma _d$ are negatively correlated to $N_f$. The most sensitive parameter is $\beta$. For the chemo-tactic parameters, the chemo-attractant parameter $\chi$ is more sensitive than the chemo-repellent parameter $\mu$.

**Table 3:** Sensitive index of the parameters used in the model.

**Parameter**	$\chi$	$\mu$	$\beta$	$\delta$	$k_{a_2}$	$k_{d_2}$	$k_{i_2}$	$\Gamma _d$
$S_j$	0.01356	−0.00013	0.019247	−0.00278	−0.00159	0.00113	0.00051	−0.00851

### Numerical simulation

We perform extensive numerical simulations with the help of MATLAB software. In our simulation, we discretize the Laplacian term and 1st order spatial derivative using central difference with mesh size $h=0.02$ and time integration is performed using forward Euler scheme with $\Delta t=10^{-6}$. We have also checked the results with smaller choices of *h* and $\Delta t$ to ensure that the obtained results are free from any numerical artifact. Results are generated upto dimensionless time $t_{\text {max}}=5$, which corresponds to a time of 5 hours in the experimental setup or the dimensional system (7).

**Figure 3:**
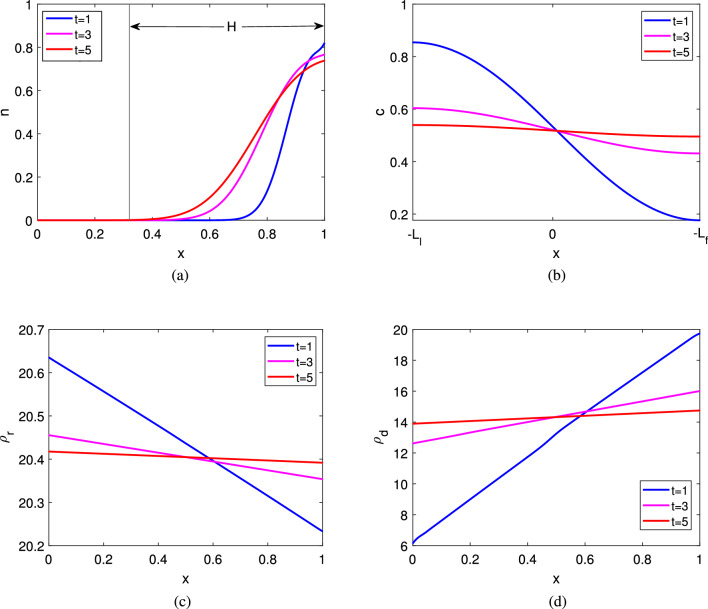
Solution for system (8-9) : (**a**) The distribution of cells in the filter, (**b**) ligand distribution in whole chamber, (**c**) distribution of normal receptors in the filter (**d**) distribution of decoy receptors in the filter at different times. Here $\beta =0.97$ and $\delta =0.952$. Other parameter values are given in Table 2.

We numerically solve system (8–9) for the cell density *n*,  the number of bound receptors $\rho _r$, the number of bound decoy receptors $\rho _d$ in the filter region, and the ligand concentration *c* in the whole Boyden’s chamber. The parameter values are mentioned in Table 2. Results are generated upto time $t_{\text {max}}=5$. We consider the number of cells in the upper well $n_0=4\times 10^{5}$ cells/cm$^3$ and chemical concentration $c_0=6\times 10^{-8}$ mol/cm$^3$ initially. We consider here the increasing rate of normal receptors $\beta =0.97$ and increasing rate of decoy receptor $\delta =0.952.$ As time increases, the cell migrates from upper well to filter. The cell density in the upper end of the filter decreases with time and the migrated area inside the filter increases with time. The monotone wave profile of cells is shown for different times $t=1,\;3$ and 5 in Fig. 3a. The ligand spreads throughout the whole chamber (see Fig. 3b). Chemical concentration in the upper well increases with time and at $t=5$, the ligand distribution becomes almost homogeneous. In Fig. 3c and d, the distribution of bound normal and bound decoy receptors is shown respectively. We denote distance travelled inside the filter at $t=5$ by *H*. We determine the value of *H* as

$$\begin{aligned} H=1-\text {Sup}\left\{ x: n(x,t=5)<\frac{1}{n_0}\right\} , \end{aligned}$$

where $n(x,t=5)$ is the cell density at time $t=5.$ We observe that *H* increases with time *t* but when the heterogeneity of the distribution of *c* decreases the rate of change of *H* decreases too.

### Variation in initial distribution

**Figure 4:**
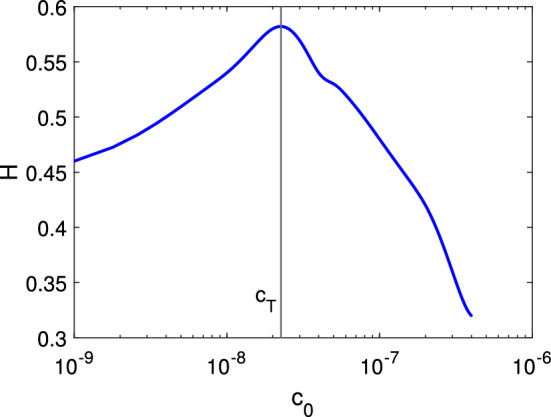
Distance travelled by leading font for different initial cell concentrations at time $t=5$. Here $\beta =0.97$ and $\delta =0.952$. Other parameter values are given in Table 2.

In Fig. 3, we fix $n_0=4\times 10^{5}$ cells/cm$^3$ and $c_0=6\times 10^{-8}$ mol/cm$^3$ and now we consider the initial chemical concentration $c_0$ and initial cell number $n_0$ as parameters. Since we have taken the initial distribution of cells for the dimensionless system (8) to be 1 in the upper end of the filter and 0 everywhere else in the filter, then $n_0$ doesn’t affect the wave profile of *n*. But $c_0$ influences the system dynamics significantly. We measure distance travelled inside the filter (*H*) for the non-dimensional model (8–9) for different initial ligand concentration $(c_0)$. We plot $c_0$ vs *H* in Fig. 4 and observe that initially with an increase in $c_0$, *H* increases but after critical threshold of $c_T=2.26\times 10^{-8},$ *H* decreases with an increase in $c_0$.

### Effect of $\beta$ and $\delta$ in cell migration

**Figure 5:**
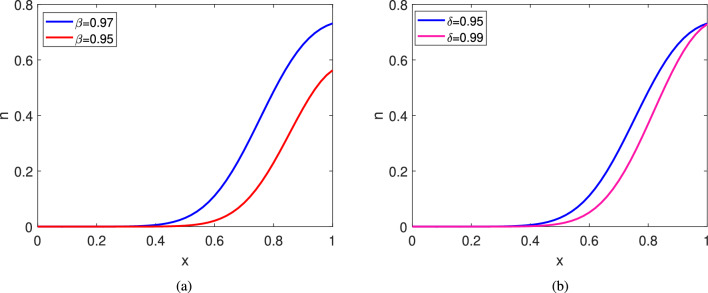
Comparison of cell’s wavefront inside filter at time $t=5$: (**a**) for different parameter values of $\beta$ with fix parameter $\delta =0.95$, (**b**) for different parameter values of $\delta$ with fix parameter $\beta =0.97$. Other parameter values are given in Table22.

Here we focus on the role of the increasing rate of normal receptors and decoy receptors $\beta$ and $\delta$ in the cell migration mechanism. The distribution of the number of bound normal receptors ($\rho _r$) increases with an increase in $\beta$ which in turn increases the chemo-attractant sensitivity. As a result, the wave-front of the migrated cells travels much deeper inside the filter. In Fig. 5a, for $\beta =0.97$, the number of cells in the filter increases $85.19\%$ more in comparison to $\beta =0.95.$ Further, an increase in $\delta$ with a fixed $\beta$, increases the distribution of decoy receptors. Therefore, cell migration is interrupted more due to additional presence of decoy receptors. For $\delta =0.99$, the number of cells in the filter decreases by $19.46\%$ in comparison to $\delta =0.95$ (see Fig. 5b). From Fig. 5a and b, we conclude that the chemo-attractant sensitivity of normal receptors influences the system dynamics much more in comparison to chemo-repellent sensitivity of decoy receptors.

### Model validation with experimental results

#### Comparison of cell movement in absence of decoy receptors

To consider the case of cell movement in absence of decoy receptors, we set all the parameters associated with decoy receptors to zero. Under this assumption, the zero initial condition of $\rho _d$ in (8d) gives only trivial solution for $\rho _d.$ This leads to the system in the filter, a three compartment coupled partial differential equations (PDE) as follows:

12a $$\begin{aligned} \frac{\partial n}{\partial t}=&-\frac{\partial K_n}{\partial x}, \qquad \qquad \quad \frac{\partial c}{\partial t}=D_c \frac{\partial ^2 c}{\partial x^2}+k_{d_1}\Gamma _r n \rho _r-k_{a_1} c n \Gamma _r \left( 1+(\beta -1) \rho _r \right) , \end{aligned}$$

12b $$\begin{aligned} \frac{\partial \rho _r}{\partial t}=&- \frac{K_n}{n}\frac{\partial \rho _r}{\partial x}+k_{a_1} c \left( 1+(\beta -1) \rho _r \right) -\left( k_{d_1} +k_{i_1}\right) \rho _r, \end{aligned}$$

where $$\displaystyle K_n=-D_n \frac{\partial n }{\partial x}+\chi n \frac{\partial \rho _r}{\partial x}.\;$$

**Figure 6:**
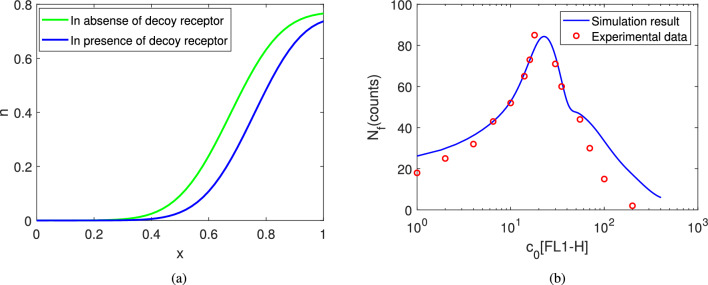
(**a**) Comparison of cell migration in presence and absence of decoy receptors at $t=5$. Here $\beta =0.97$ and $\delta =0.952$ in presence of decoy receptors and $\beta =0.97$ and $\delta =0$ in absence of decoy receptors. (**b**) Cell count in the filter for different initial cell concentrations at time $t=5.$ Here $n_0=5\times 10^{3}$ cells/cm$^3$, $\beta =0.97$, $\delta =0.952$. Experimental data is collected from Fig. 3B^8^, LN-229 cell in presence of decoy receptor DcR3. All other parameter values for (**a**) and (**b**) are given in Table 2.

System (9, 12) is subjected to same initial and boundary conditions as before. We observe that the migrated cell’s wave comes deeper inside the filter in comparison to the presence of decoy receptors (see Fig. 6a). The number of cell counts in the filter of the system (9, 12) increases by $38.43\%$ in comparison to the system (8–9). In absence of decoy receptors, cell migration is influenced by cell random movement and the chemo-attractant sensitivity of the normal receptors. But in the case of system (8–9), the decoy receptors inhibit the process of cell migration mechanism by its chemo-repellent sensitivity which results in a lesser number of cells in the filter. Roth et al.^8^ did experiments with N9 mouse microglia cells in a 48-well micro chemotaxis chamber where they had observed that the migration of microglia was substantially increased after the addition of 100 units/ml CD95L in the DMEM medium but the motility was checked in presence of 150 units/ml DcR3 (see Fig. 6 in^8^). We implement our mathematical model to study the movement of glioma cells in the presence of normal receptors CD95, ligand CD95L and decoy receptors DcR3^8^. Our simulation results are in agreement with the experimental results reported in^8^.

#### Effect of CD95L in the Filter’s Cell-counts

Roth et al.^8^ performed a flow cytometry and the immunoblot analysis to understand how DcR3 obstructs CD95L-induced mechanisms including cell apoptosis, cell migration, cell infiltration and so on. In Fig. 3B of^8^, the number of DcR3 transfected glioma cells in the cell lines LN-18, LN-229, and U373MG with varying concentrations of CD95L is obtained. In the experimental result, we observe that the count of cells increases with an increase in concentration of CD95L initially but after a certain threshold in the concentration ($c_0[\text {FL1-H}]=18$), the count of cells decreases with an increase in concentration of CD95L. The experimental data is provided in supplementary data. We plot the experimental data in Fig. 6b with red ‘o’. In the *x*-axis, FL1-H scale denotes the relative concentration of CD95L. Similar pattern is also observed in our numerical simulation. We consider $n_0=5\times 10^3$ cells/cm$^3$ and chemical concentration $c_0$ as a variable. In order to match the scale with experimental results, we re-scale the initial concentration $c_0$ with a scaling factor $10^9.$ We plot the numerical solution $N_f$ with varying $c_0$ in Fig. 6b with blue solid curve. Figure 6b shows that $N_f$ increases with an increase in initial ligand concentration, but after a certain threshold of ligand concentration ($c_0[\text {FL1-H}]=22.58$), the relationship is reversed, with excess ligand concentration the value of $N_f$ decreases. Figure 6b shows that the numerical results are in close agreement with the experimental results in^8^.

## Discussion

Cell migration is a directed movement of a single cell or a group of cells in response to chemo-tactic signals. Receptor-ligand interaction is a major class of protein-protein interactions and it activates chemokine’s downstream effectors which are responsible for chemo-tactic cell signalling. This chemo-tactic signalling does not always act as an attractant. A few receptors in the TNFRSF family have been identified as decoy receptors that recognize chemokines but do not induce cell migration. Roth et al.^8^ did an experiment in a 48-well micro chemotaxis chamber which suggests that cell migration of gliomas cells in presence of additional decoy receptors DcR3 is significantly decreased when compared to migration in presence of only normal receptors CD95. In this work, we have extended the classic Keller-Segel model^28^ to study cell migration of glioma cells in response to normal receptor CD95’s chemo-attractant sensitivity and decoy receptor DCR3’s chemo-repellent sensitivity. Here, we propose a mathematical model which consists of four partial differential equations that describe the spatio-temporal dynamics of cells, normal receptors, ligands and decoy receptors. We consider the Boyden chamber assay as a domain of our spatio-temporal model here but our model is not assay-specific except for the boundary conditions and we have validated this model with experimental results obtained from a 48-well micro chemotaxis chamber. We assume that initially cells and ligands are present only on the upper and lower well of the Boyden chamber respectively. The dynamics of cells, normal receptors and decoy receptors are presented upto $$t_{max}=5 $$ in the filter section. The cell motility in our model is influenced by three factors: (1) cell’s random movement, (2) chemo-attractant sensitivity of normal receptors, (3) chemo-repellent sensitivity of decoy receptor. The migrated cells move from upper well to the filter section. With the advancement of time, the spatial heterogeneity of the ligand, normal receptor and decoy receptor’s distribution decreases. We observe that the initial ligand concentration plays an important role in cell migration. As seen in Fig. 4, cell migration increases with an increase in initial ligand concentration but after a certain threshold point of ligand concentration ($$c_T$$), the relationship is reversed. One possible explanation for this phenomenon is that at high ligand concentration, the receptors on the cell surface become over-stimulated, leading to a depletion of free receptors available for binding^40,41^. This can interfere with the ability of the cells to respond to directional cues from the ligand and may even result in a loss of sensitivity to the signal. Additionally, excess ligands can potentially bind to receptors that are not involved in directing cell migration, leading to a loss of directionality^42^. This can ultimately result in a decrease in the intensity of cell movement, as the cells become less efficient at responding to the ligand signal.

Our research here successfully captures the dynamics of both the chemo-attractant activity of the normal receptors as well as the chemo-repellent activity of the decoy receptors. With an increasing rate of the normal receptors ($\beta$), the number of migrated cells in the filter increases whereas with an increasing rate of the decoy receptors ($\delta$), the number of migrated cells in the filter decreases. We also investigate the system dynamics in the absence of decoy receptors. Figure 6a shows that the wave front curve of migrated cells is higher in the absence of decoy receptors when compared in the presence of decoy receptors. In other words, in the presence of decoy receptors, the number of migrated cells in the filter decreases. Our results support Roth’s experiments^8^ and we also validate our simulation results with the number of cells in the filter with changes in the initial ligand concentration. The sensitivity analysis is carried out to understand the role of important model parameters in the cell migration. Our analysis predicts that the chemo-attractant sensitivity of normal receptors is stronger than chemo-repellent sensitivity of decoy receptors.

We observe here how a cell migration model is deployed to predict the movement of glioma cells due to reaction kinetic mechanism between CD95 receptors and ligand CD95L as well as compute the distance travelled by cells inside filter. A systemic approach of exploring cell migration due to decoy receptors and studying few aspect of its movement through computational models and validating cell migration with experimental results is the strength of our study here. This work is a preliminary and a crucial step toward future in understanding the influence of decoy receptors on other mechanisms such as cell apoptosis, cell adhesion, cell signaling, and so on. Our analysis of chemotactic cell response due to receptors will have significant application and open doors to future research in quantitative understanding of immunotaxis, leukocyte migration during immune response to inflammation, and chemotactic behavior of nanoswimmers in penetration to the brain to name a few^6,7,43^.

## Supplementary Information

Supplementary Information.

## Data Availability

The datasets generated and/or analyzed during the current study are available in S2.xlsx available with the manuscript as supplementary information and also in the published article^8^.
