# Author Correction: A mathematical modeling technique to understand the role of decoy receptors in ligand-receptor interaction

**DOI:** 10.1038/s41598-023-35682-8

**Published:** 2023-06-05

**Authors:** Subrata Dey, Aditi Ghosh, Malay Banerjee

**Affiliations:** 1 Department of Mathematics and Statistics, Indian Institute of Technology Kanpur, Kanpur, Uttar Pradesh India; 2 Texas A&M University Commerce, Commerce, TX 75429 USA

Correction to: *Scientific Reports* 10.1038/s41598-023-33596-z, published online 21 April 2023

The original version of this Article contained an error in Affiliation 2, which was incorrectly given as ‘Department of Mathematics, Texas A&M Commerce, Commerce, TX, USA’.

The correct affiliation is listed below:

‘Texas A&M University Commerce, Commerce, TX, 75429, US.’

The original Article has been corrected.

