# Supplementary material for: A mathematical modeling technique to understand the role of decoy receptors in ligand-receptor interaction
Source: Sci Rep. 2023 Apr 21;13:6523. doi: 10.1038/s41598-023-33596-z (PMC10121693; doi:10.1038/s41598-023-33596-z)
Supplement: Supplementary file 1 — Supplementary Information. [file 41598_2023_33596_MOESM1_ESM.pdf]

Supplementary information S1 for "A mathematical modeling technique to understand the role of decoy receptors in ligand-receptor interaction"

Subrata Dey, Aditi Ghosh, Malay Banerjee

Correspondence to: Aditi.Ghosh@tamuc.edu

Sensitivity Analysis

Here we obtain the sensitivity of the number of migrant cells in the filter i.e. $N_f = n_0 \int_0^1 n(x,\tau)dx$ at $\tau = 5$ to some important parameters of the system (8). We use similar technique as in [1, 2, 3] for our PDE model. Let $y = (y_1, y_2, y_3, y_4)^T = (n, c, \rho_r, \rho_d)^T$ and $p = (p_1, p_2, p_3, p_4, p_5, p_6, p_7, p_8)^T = (\chi, \mu, \beta, \delta, k_{a_2}, k_{d_2}, k_{i_2}, \Gamma_d)^T$. The sensitivity index S_j of the parameter p_j is defined as

$$S_j = \frac{p_j \int_0^1 \frac{\partial y_1(x,\tau)}{\partial p_j} dx}{\int_0^1 y_1(x,\tau)dx} \quad \text{for } j = 1, \cdots, 8.$$

To find $\frac{\partial y_1(x,\tau)}{\partial p_j}$, we write our model (8) in a matrix form as

$$\frac{\partial y}{\partial t} = \mathcal{F}(y, p, y_x, y_{xx}) \tag{1}$$

where $\mathcal{F}(y, p, y_x, y_{xx}) \equiv (\mathcal{F}_1, \mathcal{F}_2, \mathcal{F}_3, \mathcal{F}_4)^T$ are the reaction terms in the right-hand side of (8). Differentiating (1) with respect to p_j, we have

$$\frac{\partial}{\partial t}\left(\frac{\partial y_k}{\partial p_j}\right) = \sum_{l=1}^{4} \frac{\partial \mathcal{F}_k}{\partial y_l}\frac{\partial y_l}{\partial p_j} + \sum_{l=1}^{4} \frac{\partial \mathcal{F}_k}{\partial(\frac{\partial y_l}{\partial x})}\frac{\partial(\frac{\partial y_l}{\partial x})}{\partial p_j} + \sum_{l=1}^{4} \frac{\partial \mathcal{F}_k}{\partial(\frac{\partial^2 y_l}{\partial x^2})}\frac{\partial(\frac{\partial^2 y_l}{\partial x^2})}{\partial p_j} + \frac{\partial \mathcal{F}_k}{\partial p_j} \tag{2}$$

where $k = 1, 2, 3, 4$ and $j = 1, \cdots, 8$. As an example, for $p_1 = \chi$ we obtain

$$\begin{aligned}\frac{\partial}{\partial t}\left(\frac{\partial y_1}{\partial \chi}\right) = &\left(\mu\frac{\partial^2 y_4}{\partial x^2} - \chi\frac{\partial^2 y_3}{\partial x^2}\right)\frac{\partial y_1}{\partial \chi} + D_n\frac{\partial^2}{\partial x^2}\left(\frac{\partial y_1}{\partial \chi}\right) - \left(\chi\frac{\partial y_3}{\partial x} - \mu\frac{\partial y_4}{\partial x}\right)\frac{\partial}{\partial x}\left(\frac{\partial y_1}{\partial \chi}\right) - \\ &\chi\frac{\partial}{\partial x}\left(\frac{\partial y_3}{\partial \chi}\right)\frac{\partial y_1}{\partial x} + \mu\frac{\partial}{\partial x}\left(\frac{\partial y_4}{\partial \chi}\right)\frac{\partial y_1}{\partial x} - \chi y_1\frac{\partial^2}{\partial x^2}\left(\frac{\partial y_3}{\partial \chi}\right) + \mu y_1\frac{\partial^2}{\partial x^2}\left(\frac{\partial y_4}{\partial \chi}\right) - \\ &\left(\frac{\partial y_1}{\partial x}\right)\left(\frac{\partial y_3}{\partial x}\right) - y_1\frac{\partial^2 y_3}{\partial x^2},\end{aligned} \tag{3a}$$

$$\begin{aligned}\frac{\partial}{\partial t}\left(\frac{\partial y_2}{\partial \chi}\right) = &D_c\frac{\partial^2}{\partial x^2}\left(\frac{\partial y_2}{\partial \chi}\right) + \Big(k_{d_1}\Gamma_r y_3 - k_{a_1}y_2\Gamma_r\left(1 + (\beta-1)y_3\right) - k_{a_2}y_2\Gamma_d(1 + (\delta-1)y_4) + \\ &k_{d_2}\Gamma_b y_4\Big)\frac{\partial y_1}{\partial \chi} - \Big(k_{a_1}y_1\Gamma_r\left(1 + (\beta-1)y_3\right) + k_{a_2}y_1\Gamma_d(1 + (\delta-1)y_4)\Big)\frac{\partial y_2}{\partial \chi} \\ &+ \Big(k_{d_1}\Gamma_r y_1 - k_{a_1}y_2y_1\Gamma_r(\beta-1)\Big)\frac{\partial y_3}{\partial \chi} + \Big(k_{d_2}\Gamma_b y_1 - k_{a_2}y_2y_1\Gamma_d(\delta-1)\Big)\frac{\partial y_4}{\partial \chi},\end{aligned} \tag{3b}$$

$$\begin{aligned}\frac{\partial}{\partial t}\left(\frac{\partial y_3}{\partial \chi}\right) = &-\frac{\partial}{\partial y_1}\left(\frac{K_n}{y_1}\right)\frac{\partial y_3}{\partial x}\frac{\partial y_1}{\partial \chi} + \Big(k_{a_1}\left(1 + (\beta-1)y_3\right)\Big)\frac{\partial y_2}{\partial \chi} + \Big(k_{a_1}(\beta-1) - \\ &(k_{d_1} + k_{i_1})\Big)\frac{\partial y_3}{\partial \chi} + \frac{D_n}{y_1}\frac{\partial y_3}{\partial x}\frac{\partial}{\partial x}\left(\frac{\partial y_1}{\partial \chi}\right) - \left(\frac{K_n}{y_1} + \chi\frac{\partial y_3}{\partial x}\right)\frac{\partial}{\partial x}\left(\frac{\partial y_3}{\partial \chi}\right) +\end{aligned}$$

$$\left(\mu\frac{\partial y_3}{\partial x}\right)\frac{\partial}{\partial x}\left(\frac{\partial y_4}{\partial \chi}\right)-\left(\frac{\partial y_3}{\partial x}\right)^2, \tag{3c}$$

$$\frac{\partial}{\partial t}\left(\frac{\partial y_4}{\partial \chi}\right)=-\frac{\partial}{\partial y_1}\left(\frac{K_n}{y_1}\right)\frac{\partial y_4}{\partial x}\frac{\partial y_1}{\partial \chi}+\Big(k_{a_2}\left(1+(\delta-1)y_4\right)\Big)\frac{\partial y_2}{\partial \chi}+\Big(k_{a_2}\left(\delta-1\right)$$
$$-\left(k_{d_2}+k_{i_2}\right)\Big)\frac{\partial y_4}{\partial \chi}+\left(\frac{D_n}{y_1}\frac{\partial y_4}{\partial x}\right)\frac{\partial}{\partial x}\left(\frac{\partial y_1}{\partial \chi}\right)+\left(\mu\frac{\partial y_3}{\partial x}-\frac{K_n}{y_1}\right)\frac{\partial}{\partial x}\left(\frac{\partial y_4}{\partial \chi}\right)-$$
$$\chi\frac{\partial y_4}{\partial x}\frac{\partial}{\partial x}\left(\frac{\partial y_3}{\partial \chi}\right)-\left(\frac{\partial y_3}{\partial x}\right)\left(\frac{\partial y_4}{\partial x}\right). \tag{3d}$$

Similarly, we can write the required equations for other p_j, $j=2,\cdots 8$. We solve the system (2) and (8) together with zero initial condition i.e. $\frac{\partial y}{\partial p_j}(x,0)=0$ for $0\le x\le 1$ and no flux boundary condition at both end of the filter. Using the value of $y_1(x,\tau)$ and $\frac{\partial y_1(x,\tau)}{\partial p_j}$ we obtain the the value of S_j, for $j=1,\cdots,8$.

References

[1] X Wang, D Posny, and J Wang. A reaction-convection-diffusion model for cholera spatial dynamics. *Discrete Contin. Dyn. Syst. Ser. B*, 21:2785–2809, 2016.

[2] L Arriola and JM Hyman. Sensitivity analysis for uncertainty quantification in mathematical models. *Mathematical and statistical estimation approaches in epidemiology*, pages 195–247, 2009.

[3] S Li and L Petzold. Adjoint sensitivity analysis for time-dependent partial differential equations with adaptive mesh refinement. *Journal of Computational Physics*, 198(1):310–325, 2004.
